# Micro-mechanical approaches to characterize tip growth: Insights into root hair elasto-viscoplastic properties

**DOI:** 10.1140/epje/s10189-025-00546-8

**Published:** 2026-02-09

**Authors:** Thomas Alline, Léa Cascaro, David Pereira, Atef Asnacios

**Affiliations:** UMR 7057, Laboratoire Matière et Systèmes Complexes, Université Paris Cité, CNRS, Paris, France

## Abstract

Root hairs are outgrowths of the epidermal cells of plant roots. They increase the root's exchange surface with the soil and provide it with good anchorage in the soil. Root hairs are an emblematic model of apical growth, a process also used by yeasts and hyphae to invade their environment. From a mechanical perspective, the root hair is considered as an elastic cylinder under pressure, closed by a dome that behaves like a yield fluid. We introduce here two innovative mechanical setups and protocols to characterize the mechanical properties of single growing root hairs in *Arabidopsis thaliana*. In the first setup, root hairs grow against an elastic obstacle until buckling. By measuring the critical buckling force, we determine the surface modulus and estimate the Young’s modulus of the cell wall, which aligns with previous measurements. Using a 1D elasto-viscoplastic model of root hair growth, we assess the excess pressure beyond the yield threshold (the driver of tip growth) and estimate the axial stiffness of the root hair, reflecting its elastic resistance to compression. For the second protocol, we designed a setup where a single root hair grows against a cantilever with variable stiffness, a technique adapted from our earlier work on rigidity sensing by animal cells. This method provides an independent estimate of the root hair's axial stiffness, confirming our initial findings and suggesting that this stiffness primarily involves tip compression and depends mainly on turgor pressure, at least within the low deformation regime explored.

**Graphical abstract:**

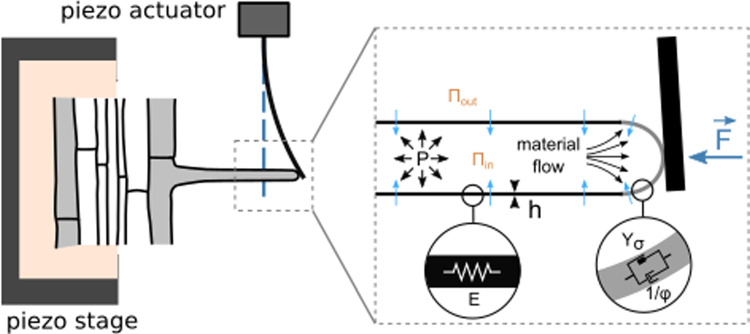

**Supplementary Information:**

The online version contains supplementary material available at 10.1140/epje/s10189-025-00546-8.

## Introduction

Root hairs are cylindrical extensions that develop from differentiated cells of the root epidermis. These highly elongated tubular structures, approximately 10 *µm* in diameter and up to a few millimetres in length [12], play a crucial role in nutrient uptake by increasing the root-soil exchange surface area (for instance by up to twofold in *Arabidopsis thaliana* [11]). To effectively grow and invade soils that present mechanical resistance, such as obstacles like rocks or compacted soil, root hair must overcome these challenges.

The invasion of soil by root hair is facilitated by turgor pressure, the motor of polarized tip growth, and by the mechanical support provided by the cell wall which is the primary component of the root hair structure. Due to its rigid structure, the cell wall maintains cell integrity under turgor pressure and protects cells from mechanical stresses. The spatiotemporal regulation of its mechanical properties is essential for cellular growth. Under isotropic turgor pressure, the regulated processes of secretion, synthesis, and modification of the cell wall at the tip lead to polar (oriented) growth of the root hair [7].

The cell wall, which is primarily composed of cellulose, hemicellulose, and pectin, is present on both the shank and tip of the cell and is crucial for tip growth. The root hair cell wall shows randomly oriented cellulose microfibrils at the tip and a weak alignment of cellulose microfibrils along the longitudinal axis in the shank [1, 15]. Measuring the mechanical properties of the root hair cell wall is thus vital for understanding the complex process of cell growth and soil invasion.

Many studies have been devoted to investigating tip growth in resistant media, but with two main strategies. On the one hand, the analysis of tip growth as a whole in media of well calibrated mechanical properties, mainly for pollen tubes and root hairs [17, 20]. On the other, measurements of specific mechanical parameters of different tip-growing species. In particular, measurements of the Young’s modulus of the cell wall have been reported in various tip-growing systems, such as pollen tubes, hyphae, and fission yeast, subjected to bending or buckling experiments [4, 13, 16]. For root hair cells, local measurements of the Young’s modulus and stiffness have been conducted using atomic force microscopy (AFM) [10, 21], and we have previously developed a flexion setup to measure the surface modulus of the cell wall of a growing root hair [18].

In this context, we present here two original mechanical setups and protocols to characterize the mechanical properties of single growing root hair of *Arabidopsis thaliana*. In the first setup, root hairs are allowed to grow against an elastic obstacle until buckling occurs. By measuring the critical force at buckling, we determine the surface modulus and estimate the Young’s modulus of the cell wall, which aligns well with values obtained from the flexion protocol [18]. We then use a 1D elasto-viscoplastic model of root hair growth to retrieve the excess pressure beyond the yield threshold (*i.e.* the motor of tip growth) as well as an estimation of the axial stiffness of the root hair, *i.e.,* its elastic axial resistance to compression. For the second experimental protocol, we designed an original setup to grow a single root hair against a cantilever of variable apparent stiffness, a technique adapted from a setup we initially developed for characterizing rigidity sensing by single animal cells [2, 14]. This protocol allows us to get an independent estimation of the apparent axial stiffness of root hair, confirming the first measurement and suggesting that this apparent root hair stiffness mainly involves root hair tip compression and depends thus primarily on turgor pressure, at least in the explored low deformation regime.

### Buckling under its own load: insight into root hair cell wall elasticity

To measure the Young’s modulus of the cell wall of a growing root hair, we relied on a buckling experiment combining a technique previously used on individual animal cells [5, 14] and a buckling protocol used for yeast [13].

**Fig. 1:**
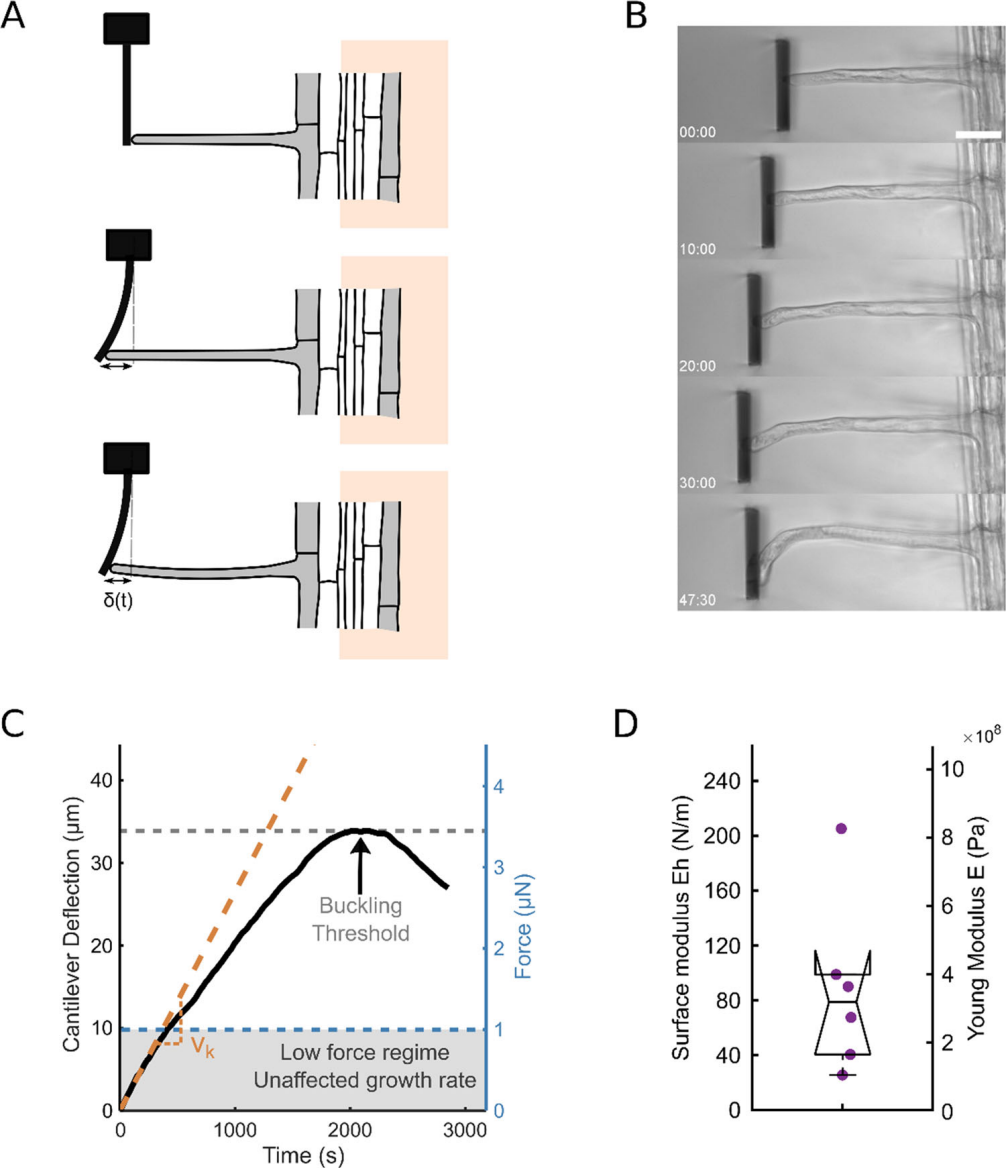
Root hair growth against a glass cantilever. **a** Schematic representing the buckling experiment. The growing root hair progressively deflects the microplate and finally buckles. **b** Successive images of a root hair growing against a microplate of stiffness *k* = 101.8 *nN*/*µm* (scale bar: 40 *µm*). The timestamp shows the time elapsed since the contact time between the root hair and the microplate. The full movie is included in the supplementary materials. **c** Deflection of the microplate by a growing root hair over time. The corresponding level of generated force is represented on the right axis. The deflection reaches a maximum when the root hair buckles under the load applied by the microplate. The orange dotted line visualizes the linearity of the elongation curve in the low-force regime, below 1 *µN*. For this experiment $v_{0} = 1.9\;\mu {\mathrm{m}}{/}\min$ and $v_{k} = 1.6\;\mu {\mathrm{m}}{/}\min$. **d** Boxplot showing the distribution of the measured surface modulus (left axis) and Young’s modulus with $h$ taken as $h = 250\;{\mathrm{nm}}$ (right axis) (*n* = 6)

*Arabidopsis* roots and root hairs were grown on 1/2 MS agar medium using a microfluidic-like system previously described [19]. This device enables high-resolution imaging under a microscope. Prior to the experiment, parts of the agar were removed to allow the root hairs to grow freely in liquid 1/2MS medium, meanwhile the root itself remained mechanically stabilized in agar to minimize drift during force application. To apply forces to the growing root hair tip, we designed a custom flexible glass microplate that functions as a cantilever of calibrated bending stiffness ${k}_{ext}$. While growing, the root hair progressively deflects the cantilever, thereby self-imposing a force $F\left(t\right)={k}_{ext} \delta \left(t\right)$ exerted on its tip, with $\delta \left(t\right)$, the cantilever deflection (Fig. 1a–c). This deflection, and thus, the force supported by the root hair, increases over time until it reaches a maximum (Fig. 1c). This maximum corresponds to the critical buckling force ${F}_{c}$. Like many tip-growing cell [3, 13], the root hair buckles under the force generated by its own growth against an obstacle, and buckling is visible in the last image of Fig. 1b.

For now, we use the critical buckling force Fc to estimate the surface modulus as well as the Young's modulus of the cell wall. The root hair is considered as a hollow cylinder of radius $R$, length $L$ and thickness $h$, with one end fixed (the root hair basis clamped to the root) and the other end free to move laterally (the root hair tip in contact with the glass plate). In these conditions, the critical force ${F}_{c}$ is related to the Young modulus $E$ and the thickness $h$ of the cell wall by ${F}_{c}=\frac{{\pi }^{3}h{R}^{3}E}{(2{L)}^{2}}$. The root hair radius and length are measured on the microscope images. We can then determine the cell wall surface modulus through the expression $Eh=\frac{4{k{\delta }_{c}L}^{2}}{{\pi }^{3}{R}^{3}}$ with ${\delta }_{c}$ the maximum deflection corresponding to ${F}_{c}$. We measure $Eh=88$ ± $26$ N/m (± SEM, *n* = 6). To estimate the cell wall Young’s modulus, we assumed the cell wall’s thickness to be $h=250\;nm$. This estimate is based on electron microscopy measurements of wild-type root hairs [1]. This gives a Young’s modulus $E=350$ ± $105\;MPa$ (± S.E.M, *n* = 6). These values are in good agreement with the values we previously measured on wild-type root hair through a bending experiment, with $Eh=113$ ± $24$ N/m and $E=453$ ± $46\;MPa$ [18]. This agreement between values obtained by two independent methods validates both the values obtained for Eh and E for the root hair wall, as well as the method of measuring Eh through the self-buckling experiment. Interestingly, the value of E we measure for single root hair is close to the Young’s modulus of wild-type pollen tube measured at 350 MPa using fluid flow to bend the cell [16], as well as to that of fission yeast estimated at 101 MPa through buckling [13].

In order to get more insight into the mechanical properties of the root hair, we will now consider a 1D elasto-viscoplastic model to analyse root hair growth, first when freely elongating in a liquid medium, and secondly when growing against an elastic obstacle.

### Root hair free growth rate

**Fig. 2:**
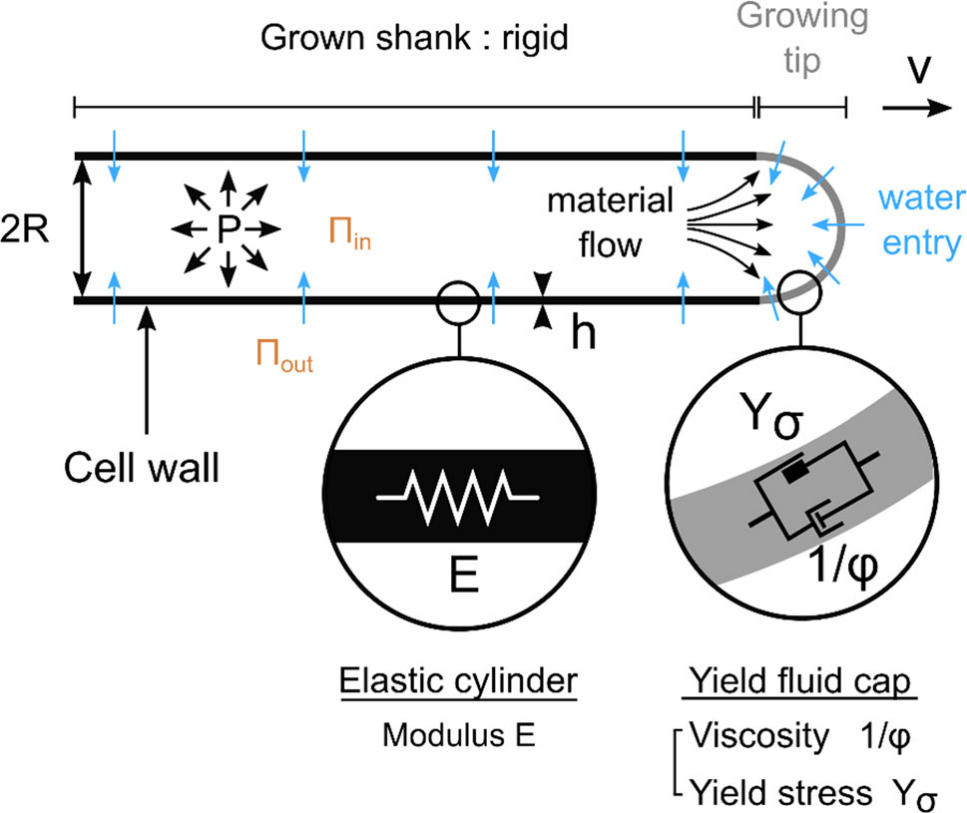
Root hair growth model. Schematic representing a root hair with a focus on the structural and mechanical parameters involved in the elasto-viscoplastic model describing its elongation and growth. The root hair is composed of a pressurized tube with a shank surrounded by an elastic cell wall. At the tip, the cell wall is described as a viscoplastic material. The turgor pressure P drives the growth at a speed v and only the tip is elongating. The root hair radius is noted R, and the thickness of the cell wall is denoted h. Y and ϕ represent the physical parameters of the viscoplastic growth model. E represents the cell wall Young modulus. Water flows inside the root hair because of an imbalance of water potential

As detailed in [6], the 1D elasto-viscoplastic model of tip growth decomposes the root hair deformation into an elastic (reversible) and a viscoplastic (irreversible) components. Indeed, from the mechanical point of view, the root hair can be seen as an elastic cylinder with a yield fluid cap (Fig. 2). For a freely growing root hair, its current length can be expressed as:

1 $$L={L}_{0}+\Delta L(P,{L}_{0})$$

${L}_{0}$ corresponds to the plasmolyzed root hair cell length, i.e., when the turgor pressure $P={P}_{in}-{P}_{out}$ is set to zero (deflated root hair). $\Delta L\left(P,{L}_{0}\right)$ represents the change in length -the elastic elongation- of the root hair when it is put under pressure. The longitudinal stress in the root hair wall due to turgor pressure is given by ${\sigma }_{L}=\frac{PR}{2h}$, the subsequent wall strain as ${\epsilon }_{L}=\frac{{\sigma }_{L}}{E}=\frac{PR}{2Eh}$, leading to $\Delta L\left(P,{L}_{0}\right)={L}_{0} {\epsilon }_{L}=\frac{{L}_{0}PR}{2Eh}$ and:

2 $$L={L}_{0}\left[1+\frac{PR}{2Eh}\right]$$

with $R, h, E$ , respectively, the radius, thickness, and elastic modulus of the root hair wall.

Irreversible growth corresponds to increasing ${L}_{0}$ by adding new material to the cell wall at the tip of the root hair, taken into account in the model by the viscoplastic, irreversible, component. In contrast, the reversible component stands for the pressure-driven elastic extension.

The overall rate of free elongation of the root hair expresses as:

3 $${v}_{e}=\frac{dL}{dt}=\frac{d{L}_{0}}{dt}\left[1+\frac{PR}{2Eh}\right]+\frac{{L}_{0}R}{2Eh}\frac{dP}{dt}$$

The term $\frac{{L}_{0}R}{2Eh}\frac{dP}{dt}$ corresponds to root hair swelling due to pressure increase. In steady-state elongation, this term would imply a continuous increase of $P$. Thus, considering turgor pressure as constant during free steady-state root hair growth, $\frac{dP}{dt}=0$ and the rate of root hair elongation writes:

4 $${v}_{e}={v}_{g}^{0}\left[1+\frac{PR}{2Eh}\right]$$

with ${v}_{g}^{0}=\frac{d{L}_{0}}{dt}$ the free growth rate corresponding to addition of material to the root hair wall. This newly added material induces an increase of the relaxed length of the root hair (plasmolysed length). $\frac{PR}{2Eh}$ is an additional component to the elongation rate due to pressure-driven wall elastic extension. With typically $P\approx 2\;bars$, $R\approx 5\;\mu m$ and $Eh\approx 100\;\frac{N}{m},$ , this elastic component seems to be negligible, with $\frac{PR}{2Eh}\approx {5.10}^{-3}$. To test this prediction, building on Eq. (2), we measured the change of root hair length after plasmolysis. Over 14 root hairs, we found $\frac{PR}{2Eh}\approx {(6\pm 1).10}^{-3}$, confirming that the elastic correction to the growth rate is negligible. Thus, in free growth, e.g. in a liquid medium with no obstacles, the observed rate of root hair elongation essentially reflects the growth rate:

5 $${v}_{e}\approx {v}_{g}^{0}=l\varphi \left({\sigma }_{L}-{Y}_{\sigma }\right)$$

where $l, \varphi , {\sigma }_{L}\text{ and}\;{Y}_{\sigma }$ are the typical length of the growth zone (~ the tip), the extensibility of the cell wall in this zone (inverse of a viscosity), the longitudinal stress in the wall (along the root hair axis), and the yield stress over which the tip flows, respectively. Equation (5) is simply the stress–strain relationship of a yield stress liquid.

### Growth against an elastic obstacle

When growing against an elastic obstacle like the glass microplate of Fig. 1b, the root hair is submitted to an opposing, compressive, force $F\left(t\right)={k}_{ext}\delta \left(t\right)$, with ${k}_{ext}$ the stiffness and $\delta \left(t\right)$ the deflection of this external spring. This force will lead to indentation of the root hair tip, as well as to reduction of the longitudinal stress in the root hair wall leading to a compression of the shank and a decreased growth rate. In the following subsections we will detail these three effects of the externally applied force.

#### Apparent stiffness of the root hair and compression-based decrease of the elongation rate

As long as buckling has not occurred, the deflection of the cantilever is equal to the elongation of the growing root hair, and therefore, its derivative (the slope of the curve of Fig. 1c) gives the elongation rate of the root hair. Here, we consider a 1D elasto-viscoplastic model to analyse the mechanics of root hair growth against an elastic obstacle to get insight into the mechanical properties of the root hair.

First, the force applied at the tip will lead to its flattening, the local mechanical equilibrium being reached when the tip-cantilever contact area $\pi {a}^{2}$ is such that $F=\pi {a}^{2}P$, the force then compensating for the action of the pressure on the flattened contact area of radius $a$. Considering the root hair tip roughly as an inflated soft hemisphere of radius $R$, its indentation by a glass microplate (the cantilever) resembles that of a Hertz contact between a sphere and a semi-infinite plane. For an indentation $I$ small as compared to $R$, the radius $a$ of the contact area is then given by:

6 $$a\sim \sqrt{RI}$$

The force applied by the cantilever can then be expressed as:

7 $$F=\pi {a}^{2}P=\pi RPI$$

As already observed for other plant cells [8], the root hair tip then behaves like a spring of apparent stiffness:

8 $${k}_{p}=\frac{F}{I}=\pi RP$$

With typically $P\approx 2\;bars$ and $R\approx 5\;\mu m$, one gets ${k}_{p}\approx 3\;\mu N{/}\mu m$. Thus, the buckling force being about 3 *µN*, in the buckling-free regime $I<1\;\mu m\ll R$ ensuring the validity of the Hertz model and the linearity of the tip response.

Beyond tip indentation, the force *F* applied along the axis of the root hair also generates a compressing longitudinal stress in the root hair wall. Outside the growth zone at the tip (of typical length $R$), the root hair can essentially be considered as a hollow cylinder with a radius *R* ~ 5 *µm*, a length $L,$ a thickness *h* ~ 250 nm, and composed of an elastic material (the cell wall) with a Young Modulus *E* ~ 400 MPa. When an axial force *F* is applied on the section of the cylinder over an area ~ $2\pi Rh$, it leads to a longitudinal stress:

9 $${\sigma }_{L}(F)=\frac{F}{2\pi Rh}=E\frac{\Delta L}{ L}$$

With $\Delta L$ the subsequent decrease in cell length, and $\frac{\Delta L}{ L}$ the corresponding strain. This relationship can be expressed in terms of force and elongation:

10 $$F=\frac{2\pi RhE}{ L }\Delta L$$

Thus giving the apparent root hair shank stiffness:

11 $${k}_{E}=\frac{2\pi REh}{ L}$$

With typically $Eh\approx 100\;N/m$, $R\approx 5\;\mu m$ and $L$, in the range 100 to 500 *µm*, ${k}_{E}$ falls within the range 6 to 30 *µN*/*µm*. Thus, ${k}_{E}$ is higher than ${k}_{p}$, the root hair tip is softer than its shank. The shank compression $\Delta L$ is smaller than the tip indentation $I$ and, with a buckling force of about 3 *µN*, in our experiments $\Delta L<I<1\;\mu m$, and represents at most a fraction of a micron. As a consequence, while ${k}_{E}$ depends in principle on the root length $L$, $\Delta L/L$ represents less than a 1% in our experiments, and ${k}_{E}$ can be considered constant.

Using Eq. (2) and taking into account tip indentation and shank compression, one can now express the root hair length when submitted to an axial compressive force:

12 $$L={L}_{0}\left[1+\frac{PR}{2Eh}\right]-\Delta L-I$$

The tip and shank act as two springs in series with $\Delta L=\frac{F}{{k}_{E}}$ and $I=\frac{F}{{k}_{p}}$. The root hair can then be seen as a whole as a spring with an effective stiffness ${k}_{cell}=\frac{{k}_{p}{k}_{E}}{{k}_{p}+{k}_{E}}$, representing the axial deformation under axial loading. In these conditions, the root hair length is reduced by $\frac{F}{{k}_{cell}}$ due to external compression:

13 $$L={L}_{0}\left[1+\frac{PR}{2Eh}\right]-\frac{F}{{k}_{cell}}$$

And, for constant *P*, the elongation becomes:

14 $$\frac{dL}{dt}=\frac{d{L}_{0}}{dt}-\frac{1}{{k}_{cell}}\frac{dF}{dt}={v}_{g}^{0}-\frac{{k}_{ext}}{{k}_{cell}}\frac{dL}{dt}$$

​where we took into account that $\frac{PR}{2Eh}\approx {(6\pm 1).10}^{-3}$ is negligible, and the fact that, before buckling, the cantilever deflection is equal to the cell elongation, $d\delta =dL.$

Equation (14) leads to:

15 $${v}_{k}=\frac{dL}{dt}=\frac{{k}_{Cell}}{{k}_{Cell}+{k}_{ext}}{v}_{g}^{0}$$

Implying a linear root hair elongation over time, but with a reduced speed due to elastic compression, which involves a direct comparison of ${k}_{Cell}$ to ${k}_{ext}$. We will use this expression later in the section dedicated to external stiffness modulation. For now, it must be noticed that (15) is only valid for a low-force regime where the irreversible growth, i.e. ${v}_{0}$, is unaffected by the compressive force. This regime is indeed observed on Fig. 1c for forces less than 1 *µN*.

#### Force-induced decrease of the root hair growth rate

As shown in Eq. (9) and as already reported for yeast [13], the force *F* externally applied on the root hair reduces the longitudinal stress in the cell wall, reducing thus the growth rate, Eq. (5) becomes:

16 $${v}_{g}\left(F\right)=l\phi ({\sigma }_{L}-{Y}_{\sigma }-\frac{F}{2\pi Rh})$$

With *R* and *h* being the radius of the root hair and its wall thickness, respectively (Fig. 2). This expression can also be written as:

17 $${v}_{g}\left(F\right)={v}_{g}^{0}\left(1-\frac{F}{2\pi Rh\left({\sigma }_{L}-{Y}_{\sigma }\right)}\right)$$

And the low-force regime corresponds in fact to forces inducing small longitudinal wall stress $\frac{F}{2\pi Rh}$ as compared to the excess stress $\left({\sigma }_{L}-{Y}_{\sigma }\right)$ driving root hair growth. Using ${\sigma }_{L}(P)=\frac{PR}{2h}$, one can express ${v}_{g}\left(F\right)$ as function of the excess (growth) pressure ${P}_{G}=P-{Y}_{p}$ with ${Y}_{p}=\frac{2h}{R}{Y}_{\sigma }$ the yield pressure:

18 $${v}_{g}\left(F\right)={v}_{g}^{0}\left(1-\frac{F}{\pi {R}^{2}{P}_{G}}\right)$$

#### Estimating root hair stiffness and growth pressure

Replacing ${v}_{g}^{0}$ by ${v}_{g}\left(F\right)$ in (4), one gets the differential equation controlling root hair elongation over time and taking into account both the elastic compression and growth reduction effects imposed by the cantilever facing the root hair:

19 $$\frac{d\delta }{dt}={v}_{k}[1-\frac{{k}_{ext}\delta }{\pi {R}^{2}{P}_{G}}]$$

Written here in terms of the cantilever deflection $\delta \left(t\right)=L\left(t\right)-{L}_{i}$, with ${L}_{i}$ the initial root hair length, when contacting the cantilever. This leads to an exponentially saturating behaviour for root hair elongation and force, at least before the onset of buckling:

20 $$\delta \left(t\right)={\tau }_{k}{v}_{k }\left(1-{e}^{-\frac{t}{{\tau }_{k}}}\right)$$

With

21 $${\tau }_{k}=\frac{\pi {R}^{2}{P}_{G}}{{k}_{ext}{v}_{k}}=\frac{\pi {R}^{2}{P}_{G}}{{k}_{s}{v}_{g}^{0}}$$

and

22 $${k}_{s}=\frac{{k}_{Cell} {k}_{ext}}{{k}_{Cell}+{k}_{ext}}$$

the apparent stiffness of the root hair and the cantilever in series.

Fitting the deflection curves with Eq. (20), we could get ${\tau }_{k}$ and ${v}_{k}$, and subsequently, the values of the excess growth pressure ${P}_{G}$ as well as the root hair stiffness ${k}_{Cell}$ through:

23 $$\left\{\begin{array}{c}{P}_{G}=\frac{{\tau }_{k}{v}_{k}{k}_{ext}}{\pi {R}^{2}}\\ \\ {k}_{Cell}=\frac{{k}_{ext}}{(\frac{{v}_{g}^{0}}{{v}_{k}}-1)}\end{array}\right.$$

For the six cells tested (Table S1), we found $${P}_{G}=(1.07$$ ± $$0.23) {10}^{5}$$ Pa ≈ 1 bar, an estimation obtained in a non-invasive way for single growing root hairs.

To estimate ${k}_{Cell}$ we first measured ${v}_{g}^{0}$ for each cell as the tip speed before the root hair enters in contact with the cantilever and then used Eq. (13) to get the apparent root hair stiffness. We found ${k}_{Cell}=0.9$ ± $0.3\;\mu N/\mu m\approx 1\;\mu N/\mu m$. This value is close to the lowest values one can estimate for ${k}_{Cell}$. As discussed previously, for the typical root hair parameters, ${k}_{p}\approx 3\;\mu N/\mu m$ and ${k}_{E}\approx 6\;\mu N/\mu m$ for the longest, and thus the softest, root hair tested, leading to ${k}_{Cell}\approx 2\;\mu N/\mu m$.

At this stage, we sought to confirm the value of ${k}_{Cell}$ using another protocol. Indeed, as observed in Fig. 1c, the cells buckle well before the force reaches its exponential saturation. The parameters of the adjustments, particularly the characteristic time ${\tau }_{k}$, are therefore poorly defined, which could lead to significant uncertainties in the estimation of ${k}_{Cell}$. Additionally, we assumed constant pressure, which may be questionable when monitoring growth over periods of about 20–30 min. Finally, the measured value of ${v}_{g}^{0}$ before contact between the root hair and the cantilever, which reflects physical characteristics of the root hair such as viscosity or yield stress, could also evolve over such long observation times.

To address these questions, we decided to conduct experiments on root hair growth under conditions of time-varying external stiffness ${k}_{ext}$. These experiments are detailed in the following section. However, it is worth noting that growth measurements are only conducted for a total of 40 s, and changes in stiffness are made in less than 0.1 s, thereby limiting any potential changes in *P* and ${v}_{g}^{0}$ during the measurement. As we will see later, the measurements are taken in the low-force regime during which growth is linear over time and Eq. (15) is valid. Using two different values of ${k}_{ext}$ then allows us to even bypass the measurement of ${v}_{0}$, making the measurements of ${k}_{Cell}$ free from the difficulties posed by the measurements through exponential growth curve fitting. Finally, working with short root hairs ($L\approx 100\;\mu m$) will allow us to keep ${k}_{E}$ high ( ${k}_{E}\approx 30\;\mu N/\mu m$) as compared to ${k}_{p}\approx 3\;\mu N/\mu m$, in order to ensure that ${k}_{Cell}\approx {k}_{p}=\pi RP$ to be able to estimate the turgor pressure $P$ directly from the measurement of ${k}_{Cell}.$

### Instantaneous modulation of the elongation rate upon variation of the obstacle effective stiffness

**Fig. 3:**
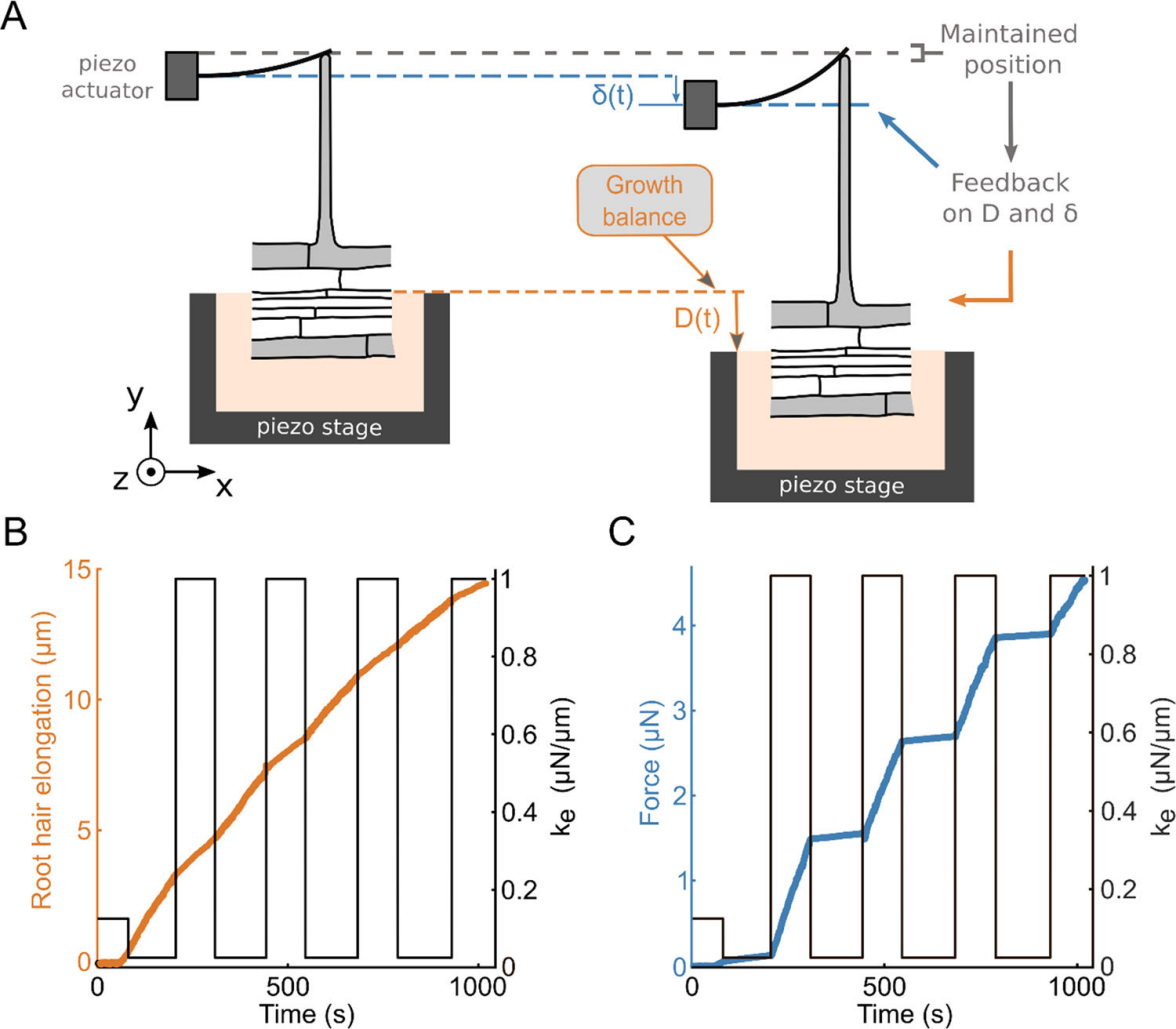
Instantaneous variations of the rates of elongation upon successive effective rigidity modulation. **a** Schematic of an iterative step of the effective rigidity setup. The position of the contact between the root hair and the microplate is maintained constant over time by a double feedback signal: $D(t)$ controls root hair elongation, and $\delta (t)$ the deflection of the cantilever (a glass microplate of calibrated stiffness), and thus the force resisting root hair elongation. **b-c** Root hair elongation and force during successive changes in effective stiffness. **b** Evolution of the root hair elongation (orange) when the cantilever effective stiffness (black) is modulated over time. **c** Evolution of the force (blue) in response to the same cantilever effective stiffness modulation (black) over time

To modulate the effective stiffness of the obstacle resisting root hair growth, we adapted a technique we have originally designed to characterize rigidity sensing by single animal cells [2, 14]. In practice, a glass microplate of calibrated stiffness is put in contact with a growing root hair as described in the previous section, but here, we use a dual feedback loop to vary at will the force–deflection relationship, *i.e.* the apparent cantilever stiffness, thus changing the effective external stiffness ${k}_{ext}$ of the obstacle facing the root hair in less than 0.1 s (Fig. 3).

The plant is mounted on a sample holder on a piezo stage which allows to control the position of the root hair base (Fig. 3a). The microplate is also mounted on a 3D micromanipulator arm on a 3D piezo actuator to control the microplate base position. A dual feedback loop is then used to control both the positions of the cantilever and the plant. The position of the contact point between the root hair apex and the microplate is measured using a position-sensitive detector. This position is maintained constant by the dual feedback despite root hair elongation during growth. To do so, the double feedback loop applies simultaneously a displacement $D\left(t\right)$ to the root holder and a displacement $\delta \left(t\right)$ to the base of the cantilever. $D\left(t\right)$ corresponds to a displacement of the root, and thus of the base of the root hair. Since the position of the contact between the root hair and cantilever tips is kept constant during the experiment, $D\left(t\right)$ is a measure of root hair elongation. Symmetrically, $\delta \left(t\right)$ corresponds to the cantilever deflection. The effective cantilever stiffness ${k}_{ext}$ is then given by the ratio between the increase in force $k\delta \left(t\right)$ and the increase in root hair length $D\left(t\right)$. Thus, ${k}_{ext}=k\frac{\delta \left(t\right)}{D\left(t\right)}$ where $k$ is the physical stiffness of the cantilever. The experimenter can adjust the effective stiffness, in real time, by modifying the ratio between $\delta \left(t\right)$ and $D\left(t\right)$ [14]. The Fig. 3b and c illustrate the response of a single root hair to successive changes in ${k}_{ext}$ between 25 and 1000 *nN*/*µm*. The elongation rate of the root hair and the rate of increase of the force change instantaneously with each change in ${k}_{ext}$. It is also noted that the elongation curve still overall resembles that of a saturating exponential as the force level increases, with a slope that decreases and thus makes it increasingly difficult to distinguish the jumps in speed. For the measurements of ${k}_{Cell}$, we therefore limited ourselves to studying the first jump, in the low-force regime, just after contact between the root hair and the cantilever.

**Fig. 4:**
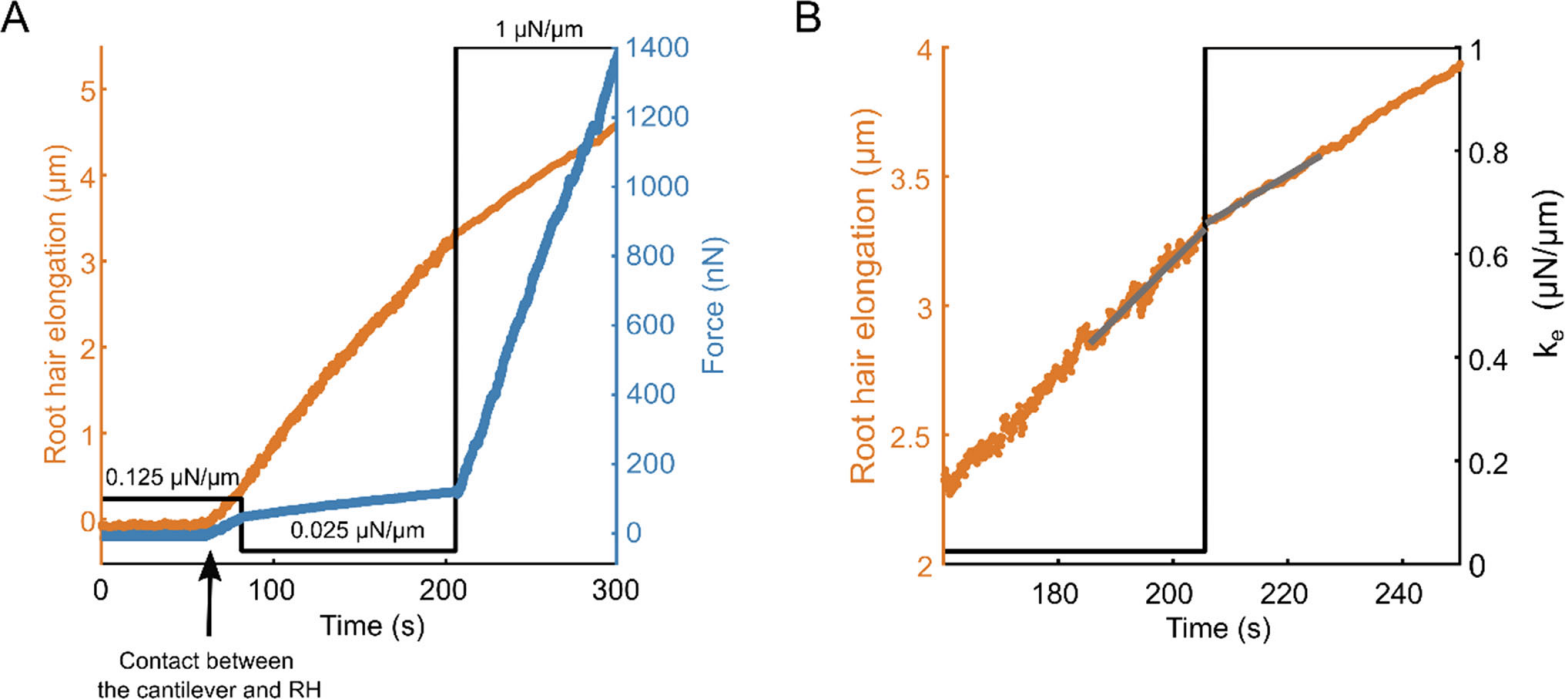
Instantaneous variation of root hair growth rate upon effective stiffness modulation. **a** Root hair elongation (orange) and force evolution (blue) over time when submitted to sudden jumps in external stiffness (black). Three successive effective stiffness values are explored successively: 0.125 *µN/µm*, 0.025 *µN/µm* and 1 *µN/µm*. Until around 80 s, the root hair is not in contact with the microplate, and no growth is measured. **b** Zoom on the evolution of the elongation rate around a change in effective stiffness from 0.025 *µN/µm* to 1 *µN/µm* (black). The grey segments are linear fits during 20 s before and 20 s after the change in stiffness

At the beginning of the experiments, a growing root hair was placed in front of a microplate, at a distance of a few micrometres, and brightfield images were taken every 2 s using a 40X objective to monitor growth. Initially, the effective stiffness was set equal to the actual physical stiffness of the microplate ($k=0.125\;\mu N/\mu m$) by applying the same displacement to both the root and the microplate base ($\delta \left(t\right)$ = $D\left(t\right))$. Once the root hair tip has reached the microplate and contact was established, the effective stiffness was set to $0.025\mu N/\mu m$ (Fig. 4a). The root hair was then allowed to grow under this low stiffness condition for a few minutes, and the effective stiffness was then increased to either 0.5 or 1 *µN*/*µm* depending on the experiment. Figure 4b shows an example of a sudden change in root hair growth rate following an increase in effective stiffness from $0.025$ to $1\;\mu N/\mu m$. To quantify this change, linear fits were performed on root base displacement data for 20 s before and after the stiffness change. For instance, in this particular experiment, the slopes show a growth speed reduction from ${v}_{0.025}=1.32\;\mu m/min$ at ${k}_{ext}=0.025\;\mu N/\mu m$ to ${v}_{1}=0.79\;\mu m/min$ at ${k}_{ext}=1\;\mu N/\mu m$. All curves were analysed for force levels < 0.4 *µN* (the force at stiffness changes was always < 0.25 *µN*), *i.e.* low as compared to the typical ~ 1 *µN* axial force necessary to significantly reduce root hair growth speed (Fig. 1c), and also implying low shank and tip deformation to ensure a linear force–deformation relationship for both springs.

To assess further le link between effective stiffness and growth rate, after the initial increase, the root anchoring in agar was checked by comparing the displacement controlled by the feedback loop and the observed root displacement on brightfield images (Figure S1).

**Fig. 5:**
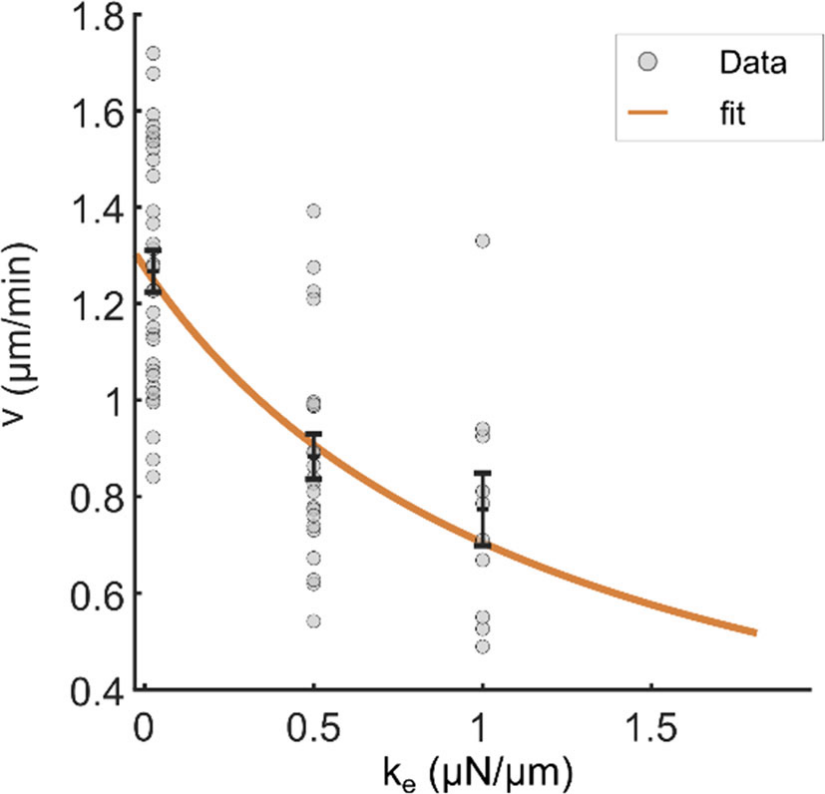
Elongation speed as a function of the cantilever effective stiffness for the cell population tested. root hair growth speed represented as a function of the cantilever effective rigidity. The black error bar represents the mean ± S.E.M for each rigidity (0.025 *µN/µm* (*n* = 32), 0.5 *µN/µm* (*n* = 22), and 1 *µN/µm* (*n* = 10)). The orange curve represents a fit of Eq. (5) to the data

Under these conditions, we consistently observed a decrease in the root hair growth rate with increasing stiffness, and the decrease was greater with larger stiffness jumps. Specifically, jumps from 0.025 to 0.5 *µN/µm* resulted in a relative speed drop of $\frac{{v}_{0.025}-{v}_{0.5}}{{v}_{0.025}}=35$ ± $5\mathrm{\%}$ (*n* = 22 cells), while jumps from 0.025 to 1 *µN/µm* caused a larger drop $\frac{{v}_{0.025}-{v}_{1}}{{v}_{0.025}}=42$ ± $4\mathrm{\%}$ (*n* = 10). This is in agreement with Eq. (15) since it fits well ${v}_{k}$ data as function of the three ${k}_{ext}$ values tested (Fig. 5), although ${v}_{k}$ displays an important variability from cell to cell.

### Estimating root hair apparent stiffness and turgor pressure from jumps in obstacle stiffness

Since measurements were taken in the low-force regime to ensure that the root hair growth rate is solely a function of ${k}_{ext}$ and not of the force *F*, Eq. (15) is valid, and we can express it for two different values ${k}_{1}$ and ${k}_{2}$ of ${k}_{ext}$:

24 $$\left\{\begin{array}{c}{v}_{1}={v}_{0}\frac{{k}_{Cell}}{{k}_{Cell}+{k}_{1}}\\ \\ {v}_{2}={v}_{0}\frac{{k}_{Cell}}{{k}_{Cell}+{k}_{2}}\end{array}\right.$$

The ratio $\frac{{v}_{1}}{{v}_{2}}=\frac{{k}_{Cell}+{k}_{2}}{{k}_{Cell}+{k}_{1}}$ of the elongation rates allows one to express the root hair stiffness ${k}_{Cell}$ as a function of the applied effective stiffness ${k}_{1}$, ${k}_{2}$ and of the measured speeds ${v}_{1}$, ${v}_{2}$:

16 $${k}_{Cell}={k}_{1}\frac{\left(\frac{{k}_{2}}{{k}_{1}}\right)-\left(\frac{{v}_{1}}{{v}_{2}}\right)}{\left(\frac{{v}_{1}}{{v}_{2}}\right)-1}$$

From the 0.25 to 0.5 *µN/µm* stiffness jumps, we found ${k}_{Cell}=2.24$ ± $0.52\;\mu N/\mu m$ (*n* = 22 cells), while jumps from 0.025 to 1 $\mu N/\mu m$ led to ${k}_{Cell}=1.62$ ± $0.44\;\mu N/\mu m$ (*n* = 10). Pooling all the data leads to ${k}_{Cell}=2.04$ ± $0.38\;\mu N/\mu m$ (*n* = 32 cells). Thus, we end up with ${k}_{Cell} \sim 2\;\mu N/\mu m$ confirming, by independent experiments and protocols, the $\sim 1\;\mu N/\mu m$ found previously for root hairs growing against regular cantilevers of constant stiffness.

Of note, we have carried out the variable stiffness experiments with a specific attention to the length of the root hairs tested. We selected short root hairs of ~ 100 *µm* in length ($L=92$ ± $8\;\mu m$ over *n* = 28 cells) to ensure a high shank stiffness ${k}_{E}\approx 30\;\mu N/\mu m$ as compared to ${k}_{p}\approx 3\;\mu N/\mu m$, in order to ensure that ${k}_{Cell}\approx {k}_{p}=\pi RP,$ to be able to estimate the turgor pressure $P$ directly from the measurement of ${k}_{Cell}.$ Given that the radius of the root hair is typically 4–5 *µm*, the measured stiffness of the root hair ${k}_{Cell}$ of approximately $2\;\mu N/\mu m$ would therefore imply a pressure *P* of about 1.6 bar, which is quite plausible and certainly in the right order of magnitude.

## Discussion

Root hairs are cylindrical outgrowth of root epidermal cells that increase the surface of exchange with the soil and help the root penetrating the soil. Root hair growth is a typical example of apical (or tip) growth. This fundamental process allows plants, yeasts, and hyphae to invade their environment. This process thus involves complex mechanical aspects and numerous associated parameters, such as turgor pressure, the elastic modulus of the cell wall, the effective viscosity, and the yield threshold of the growth zone. To enhance our understanding of apical growth and validate realistic models, it is necessary to develop experimental devices and protocols to measure these parameters in vivo.

Here, we have described a micromanipulation device and original experimental protocols for measuring mechanical parameters characterizing the apical growth of root hairs when they encounter obstacles, a situation that is quite typical for this type of cellular extensions which necessarily involves soil penetration. First, through a self-buckling experiment, we were able to measure the surface modulus (*Eh* ~ 100 N/m) and then estimate the Young's modulus of the root hair wall (*E* ~ 400 MPa). Then, by analysing the growth law of the root hairs over time, we measured the excess pressure above the yield threshold (${P}_{G}$ ~ 1 bar), as well as the apparent axial stiffness of the root hairs (${k}_{Cell} \sim 1\;\mu N/\mu m$), which presumably allows them to limit their compression during soil penetration. We then performed an independent new measurement using a variable stiffness device that we specifically developed, in particular in order to limit the time over which the measurements are performed, to avoid any potential changes in *P* and ${v}_{g}^{0}$ during the measurement, as well as to keep force at very low values to ensure linearity of the mechanical root hair behaviour. We then confirmed the relatively low value of the axial stiffness of the root hairs (${k}_{Cell} \sim 2\;\mu N/\mu m$). These results highlight the importance of taking into account elastic deformations (*i.e.* reversible deformations) in mechanically constrained growth experiments. Indeed, if the applied force varies in time, the observed elongation rate is not equal to the growth rate due to cell wall yielding.

Moreover, the measurement of short root hairs—whose shank stiffness is an order of magnitude higher than that of the tip—allowed us to obtain an in situ, non-invasive, estimate of the turgor pressure *P,* which was approximately 1.6 bar. At this stage, it is tempting to combine the values of the excess pressure ${P}_{G}$ (obtained via exponential growth) and the hydrostatic pressure *P* (estimated here assuming ${k}_{Cell}$ ~ ${k}_{P}$) to estimate the pressure growth threshold ${Y}_{p}=P-{P}_{G}$. This yields ${Y}_{p}$ = 0.6 bar, a quantity for which there is currently no measurement and thus no reference in the literature. However, caution is necessary, particularly because ${P}_{G}$ and *P* were estimated from two different cell populations, and the data in Fig. 5 clearly show that cellular variability is significant. For example, growth rates at very low external stiffness (and thus close to the free growth rate ${v}_{g}^{0}$) vary between 0.8 and 1.8 *µm/min*. Indeed, the average speed for the sample used to estimate *P* is approximately 1.3 *µm/min*, whereas it is about 1.8 *µm/min* for the cells used to measure ${P}_{G}$.

To address these challenges, we are currently working on developing techniques and experimental protocols that would allow us to obtain specific and independent measurements of the turgor pressure *P* on one hand, and the yield pressure ${Y}_{p}$ of the wall in the growth zone on the other. Furthermore, to validate the idea that the tip of the root hair acts as a spring with stiffness ${k}_{P}$, which is 10 times smaller than the typical stiffness of the rest of the cell ${k}_{E}$, it would be necessary to develop precise measurements of the deformation of the cell wall along the entire contour of the root hair.

## Material and methods

### Device preparation

The microfluidic-like system [19] used to grow the roots was fabricated using a custom-made PVC mould featuring a single channel measuring 270 *µm* in height and 1 cm in width. A 10:1 mixture of PDMS base and curing agent (Sylgard 184, Dow Corning) was poured into the mould and cured overnight at 65 °C. The cured PDMS chip was then bonded to a glass microscope slide using a plasma cleaner (Harrick Plasma, PDC-002-CE). Then, the channel was filled with ½ MS (Murashige and Skoog) medium supplemented with 5% sucrose (w/w) and 1% agar (w/w) (Duchefa, plant agar), with the pH adjusted to 5.7. Finally, a 0.5-cm-thick layer of the same medium was poured around the PDMS chip.

### Plant culture

We used *Arabidopsis thaliana* seeds expressing the fluorescent nuclear envelope marker pSUN1:SUN1-GFP [9]. Seeds were sterilized and then stratified for 48 h at 5 °C in 1 mL of ½ MS (Murashige and Skoog) medium supplemented with 5% sucrose (w/w). After stratification, the seeds were planted near the entrance of the channel on the agar-filled microfluidic device. The system was then enclosed in a Petri dish sealed with microporous tape and placed at a 45° angle relative to the vertical inside an incubator (Sanyo MLR-351H). Growth conditions consisted of a 16-h light phase at 20.5 °C and an 8-h dark phase at 17 °C, with 65% humidity.

### Cantilever preparation and calibration

The microplates were made by stretching (Narishige PB-7 puller, Japan) a glass plate, then cutting it and fusing it with a glass capillary as described in a previous study [5]. The calibration of the glass microplate consisted in calibrating it with a standard microplate as described before [5]. The microplates used in buckling experiments are placed along the vertical axis whereas in the stiffness experiments, the microplate is placed parallel to the imaged plan to ensure a correct detection of its position by the position-sensitive detector.

### Force application and data collection

Prior to the experiment, the plant and the microfluidic-like system were placed on a microscope holder. Using tweezers and a scalpel, the agar on the side of the root tip was removed and replaced with ½ MS (MS Murashige and Skoog) liquid medium supplemented with 5% sucrose (w/w), PH 5.7. The system was then observed either with a 20X0.45 NA or a 40 X 0.55 NA objective under an IX83 Olympus microscope. The root was positioned at an angle to ensure that a single root hair grew in contact with the microplate. During the buckling experiment, root hair growth and the cantilever deflection were measured using brightfield images. In the variable stiffness setup, the microplate was placed in position using a 3D micromanipulator system, ensuring that its position could be recorded by a position-sensitive detector. (S3979 one-dimensional Position Sensitive Detector Hamamatsu, France). The feedback loop allowing to modulate cantilever stiffness was adapted from a previously described experiment [14]. The positions of the piezoelectric stage and piezo actuator controlling the cantilever base position were recorded during the experiment. Bright field images were taken to assess the root hair growth direction. In all experiments, root hair elongation speed was estimated by fitting the piezoelectric position versus time.

### Root hair reduction in length upon plasmolysis

*Arabidopsis thaliana* root and root hair hair were grown in solid medium (½ MS (Murashige and Skoog) medium supplemented with 5% sucrose (w/w) and 0.5% agar (w/w) (Duchefa, plant agar), with the pH adjusted to 5.7). After root hair growth arrest, using tweezers and a scalpel, the agar on the side of the root and mature root hairs was removed and replaced with ½ MS (MS Murashige and Skoog) liquid medium supplemented with 5% sucrose (w/w), PH 5.7. The turgid root hair length was then measured on brightfield images with a 40 X 0.55 NA objective under an IX83 Olympus microscope. Then, the liquid medium was replaced by the same medium with a high mannitol concentration (> 400 mM). After visual confirmation of plasmolysis, the plasmolysed root hair length was then measured on brightfield images. The observed root hairs (*n* = 14) displayed a mean turgid length of $L=438$ ± $24\;\mu m$. The observed mean reduction in length was $L - {L}_{0}=2.2$ ± $0.5\;\mu m$ which corresponds to a strain of $\varepsilon =6.{10}^{-3}$ ± $1.{10}^{-3}$.

### Statistical analysis

The data were analysed using Matlab and FIJI. The data are presented as mean values ± s.e.m. For the values determined by a fit, the Matlab Curve Fitting tool was used and data are presented as fitted value ± half-width of the 95% confidence interval.

## Supplementary Information

Below is the link to the electronic supplementary material.

Supplementary file1 (DOCX 368 KB)

## Data Availability

Data will be made available upon reasonable request.
