# Supplementary material for: Micro-mechanical approaches to characterize tip growth: Insights into root hair elasto-viscoplastic properties
Source: Eur Phys J E Soft Matter. 2026 Feb 9;49(1-2):11. doi: 10.1140/epje/s10189-025-00546-8 (PMC12886323; doi:10.1140/epje/s10189-025-00546-8)
Supplement: Supplementary file 1 — Supplementary file1 (DOCX 368 KB) [file 10189_2025_546_MOESM1_ESM.docx]

Supplementary Material - EPJE / Topical issue ‘**Physical approaches to biological systems**’

**Micro-mechanical approaches to characterize tip growth: Insights into Root Hair Elasto-Viscoplastic Properties.**

T. Alline, L. Cascaro, D. Pereira and A. Asnacios

1. **Root anchoring**

A poorly anchored root might be displaced when force is applied at the root hair tip, leading to errors in in the measured RH elongations. To check root anchoring, we compared the displacement applied by the feedback loop to the stage holding the root (black curve of Fig. S1) with the actual displacement of the root as observed in brightfield images (red). As shown in Figure S1, the two displacements overlap very well. The mismatch was not more than 500 nm over tens of microns displacements, confirming thus excellent root anchoring.

**Figure S1**

**
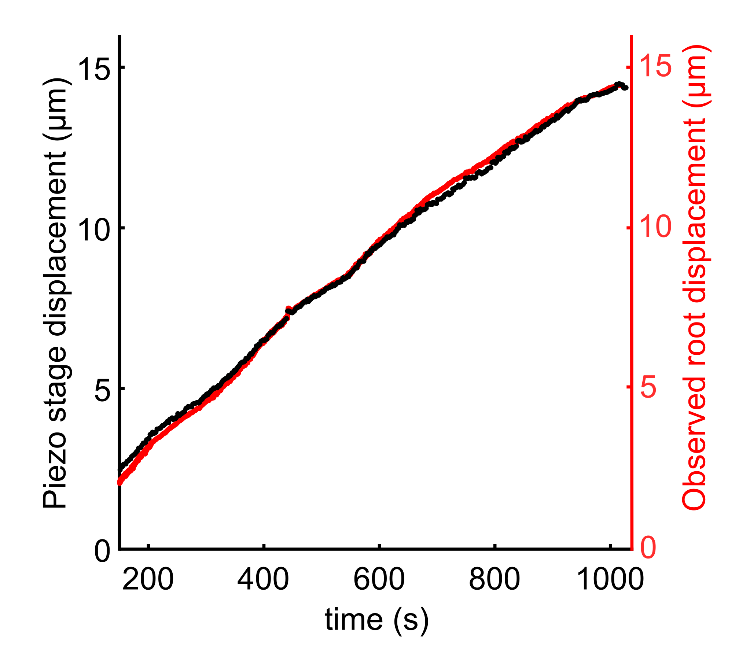
**

**Figure S1. Illustration of the good root anchorage.** During the variable stiffness experiment displayed in Figure 3, the stage displacement (black), imposed by the feedback loop on the piezo actuator controlling the root position, overlaps well with the actual root displacement (red) visualized through brightfield imaging and measured using the template matching tool of FIJI.

1. **Correlation between feedback noise and effective stiffness:**

A close look on the elongation and force curves during variable stiffness experiments reveal an uneven distribution of the noise on the signals. The RH elongation curve is noisier when the effective stiffness is low, whereas the force curve becomes noisier when stiffness is higher. This is because the feedback command is unevenly distributed among the stage displacement (RH elongation) and the cantilever base displacement (imposing deflexion and thus force). Indeed, the effective stiffness is set by the ratio between these two signals. At low effective stiffness $(k_{feedback}<k_{cantilever})$, a large fraction of the feedback command signal is devoted to elongation, whereas only a small fraction is devoted to force increase through cantilever deflection. In these conditions the feedback loop noise gets also unequally distributed between the signals. Thus, at low stiffness elongation measurements are noisier, whereas at high stiffness force measurements are noisier (Figure S2). This is particularly evident from the zoom on the elongation curve during stiffness change (Figure 4B). indeed, as visualized in Figure S2, the sum of the noises of the 2 signals is constant over time. This sum corresponds to the intrinsic noise of the feedback loop which is unevenly distributed between elongation and force depending on the chosen effective stiffness value.

**Figure S2**

**
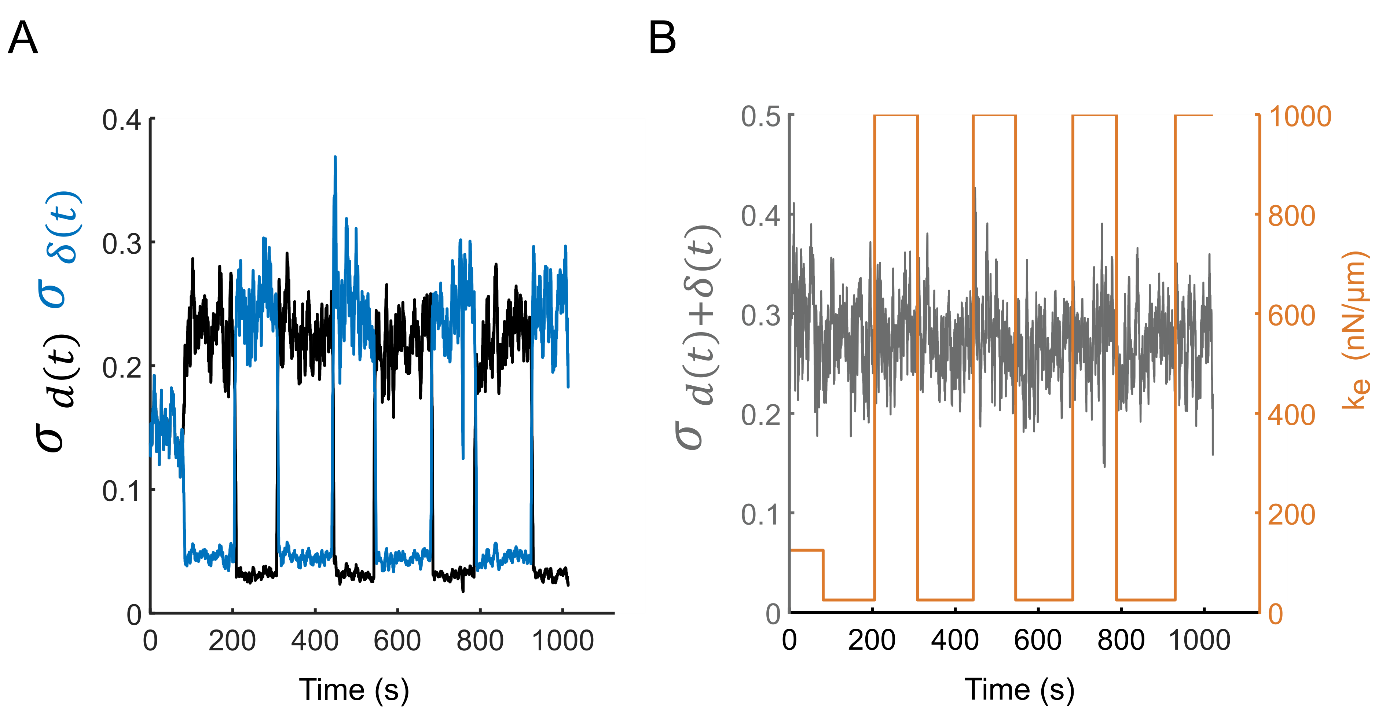
**

**Figure S2. Noise of the feedback loop.**

**A.** The black and blue curve display the standard deviation of the displacements $d(t)$ (elongation) and $\delta(t)$ (force) measured in a sliding window of 50 points (~5s) applied by the double feedback loop during the experiment showed in figure 3. **B.** The gray curve displays the standard deviation of the sum $d\left( t \right)\boldsymbol{+}\delta(t)$ measured in a sliding window of 50 points. The orange curve represents the cantilever effective stiffness.

1. **The observed root hair elongation is different from root hair growth**

**Figure S3**

**
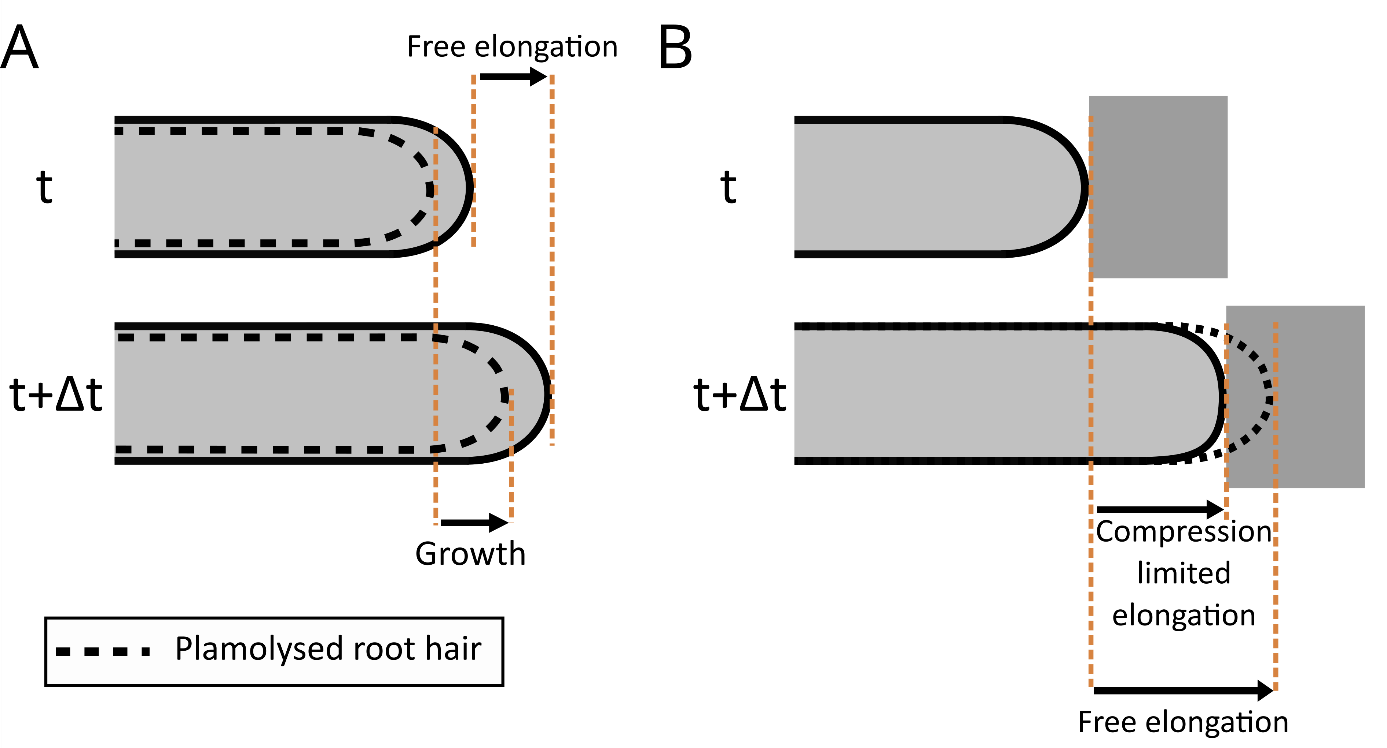
**

**Figure S3. Root hair elongation**

**A.** Schematic showing the free elongation of a root hair in a time $\text{∆t}$. The growth is defined as the irreversible increase in length due to the addition of new material in the cel wall. Growth is shown by the increase in the root hair plasmolysed length (dotted contour). **B.** Schematic showing the difference between the free elongation and the compression limited elongation which is the elongation that can be directly observed (the dotted contour stands here for the compressed tip – for illustration purpose the indentation of the tip is exaggerated. As indicated in the main text, the indentation typically of a fraction of a µm, small as compared to the root hair radius)

**Table S1**

**
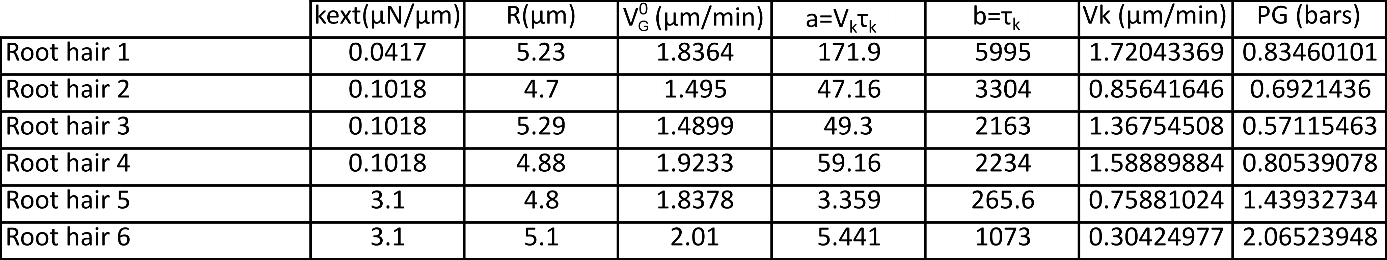
**

**Table S1.** Table showing the microplate stiffness, root hair radius, $v_{g}^{0}$, the experimental fit parameters corresponding to the equation in the main text, $\text{v}_{\text{k}}$ and the growth pressure for each of the 6 tested root hairs.
